# Supplementary figures and images for: SARS-CoV-2 Screening Testing in Schools for Children with Intellectual and Developmental Disabilities
Source: Res Sq. 2021 Jul 20:rs.3.rs-700296. Preprint. [Version 1] doi: 10.21203/rs.3.rs-700296/v1 (PMC8312901; doi:10.21203/rs.3.rs-700296/v1)

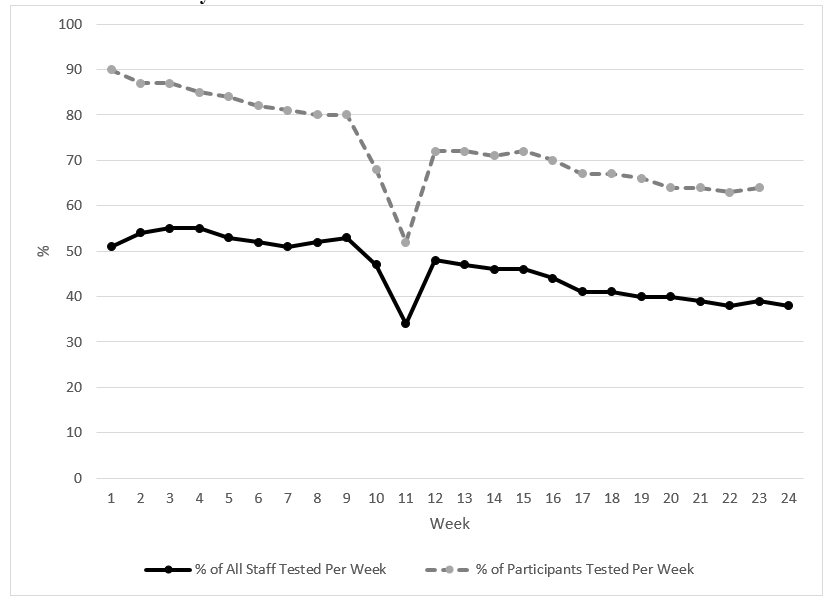

Supplement: Supplement 1 [file 5e9c49f36968c41c07f84d30.png]

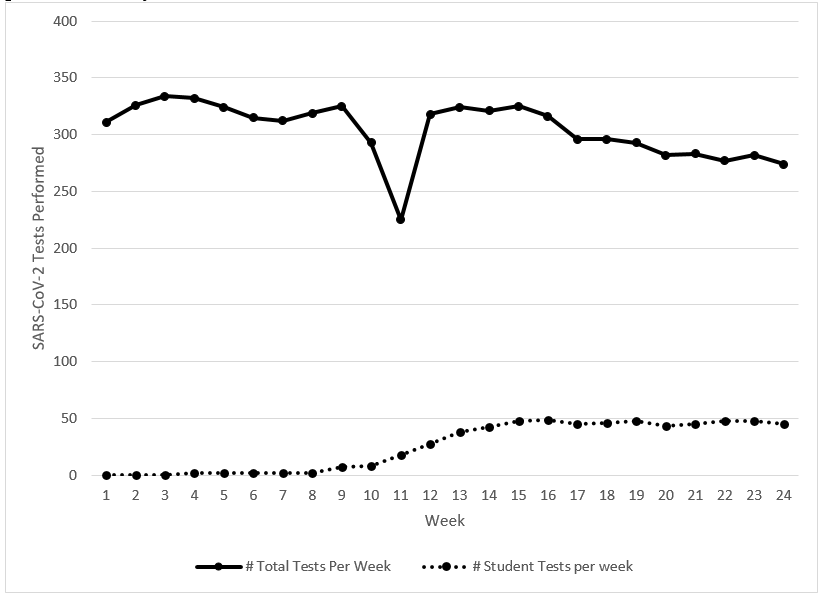

Supplement: Supplement 2 [file f649b1c0782ccd900fd87bac.png]
